# Characterizing Floral Symmetry in the Core Goodeniaceae with Geometric Morphometrics

**DOI:** 10.1371/journal.pone.0154736

**Published:** 2016-05-05

**Authors:** Andrew G. Gardner, Jonathan N. Fitz Gerald, John Menz, Kelly A. Shepherd, Dianella G. Howarth, Rachel S. Jabaily

**Affiliations:** 1 Department of Biology, Rhodes College, 2000 N. Parkway, Memphis, Tennessee, United States of America; 2 Science and Conservation Division, Department of Parks and Wildlife, Kensington, Western Australia, Australia; 3 Department of Biological Sciences, St. John’s University, Queens, New York, United States of America; Università di Pisa, ITALY

## Abstract

Core Goodeniaceae is a clade of ~330 species primarily distributed in Australia. Considerable variation in flower morphology exists within this group and we aim to use geometric morphometrics to characterize this variation across the two major subclades: *Scaevola* sensu lato (s.l.) and *Goodenia* s.l., the latter of which was hypothesized to exhibit greater variability in floral symmetry form. We test the hypothesis that floral morphological variation can be adequately characterized by our morphometric approach, and that discrete groups of floral symmetry morphologies exist, which broadly correlate with subjectively determined groups. From 335 images of 44 species in the Core Goodeniaceae, two principal components were computed that describe >98% of variation in all datasets. Increasing values of PC1 ventralize the dorsal petals (increasing the angle between them), whereas increasing values of PC2 primarily ventralize the lateral petals (decreasing the angle between them). Manipulation of these two morphological “axes” alone was sufficient to recreate any of the general floral symmetry patterns in the Core Goodeniaceae. *Goodenia* s.l. exhibits greater variance than *Scaevola* s.l. in PC1 and PC2, and has a significantly lower mean value for PC1. Clustering clearly separates fan-flowers (with dorsal petals at least 120° separated) from the others, whereas the distinction between pseudo-radial and bilabiate clusters is less clear and may form a continuum rather than two distinct groups. Transitioning from the average fan-flower to the average non-fan-flower is described almost exclusively by PC1, whereas PC2 partially describes the transition between bilabiate and pseudo-radial morphologies. Our geometric morphometric method accurately models Core Goodeniaceae floral symmetry diversity.

## Introduction

Categorization of morphological forms is an important component of comparative biology. Categories can suggest function and/or relatedness, and can be analysed in a comparative framework to assess homology, convergence, and correlation. Some categories are quite obvious, whereas others are less well delineated. Subjective morphological categories are often sufficient for taxonomic or systematic inquiries; however, new tools in population genetics, evo-devo, and next-generation molecular sequencing are detecting increasingly significant levels of genetic divergence between morphologically cryptic lineages [1, 2]. As such, methods that provide objective, quantitative alternatives to subjective categorization are becoming important, particularly when categories are not immediately obvious or variation appears continuous. Quantifying these traits provides mathematical leverage to studies of variation within populations, and to comparative character evolution and gene expression studies among species to help identify the key molecular targets of adaptive evolution. A textbook example is the adaptive radiation in Darwin’s finches. Although their beaks exhibit multiple discrete forms correlating with food sources, the overall shape of the beak is dictated by modulation of two pathways: *Bmp4* for depth, and Calmodulin for length [3, 4], operating along an axis of shape variation discerned by geometric morphometrics [5]. Studies that have shifted from binary or discrete characters to continuous characters have also opened our eyes to previously unseen patterns, as in the example of the punctuated adaptive diversification of the *Aquilegia* nectar spur in response to different pollinators (e.g., [6, 7]).

Floral symmetry provides an interesting morphological character in which to attempt quantitative modelling (e.g., [8]). The basic blueprint of floral development has been well characterized in several model systems and the essential molecular components are well conserved (e.g., [9]). The genes responsible for modifying floral forms are also increasingly being characterized in an evolutionary context in non-model systems through the hybrid field of evo-devo [10]. As adaptive floral morphology is intricately linked with pollinator interactions and environmental cues, quantifying the developmental modules responsible for floral morphology might provide insight into multiple aspects of ecology and evolution. Developing a measurable, quantitative character for floral symmetry is an important factor in clarifying modulations of the basic floral blueprint.

Geometric morphometrics is a methodology that captures quantitative measurements from the shape of complex structures, potentially uncovering patterns of shape evolution or morphological integration [11]. Geometric morphometrics differ from traditional morphometric methods in that they characterize the shape of an object as a whole, rather than in pieces, yielding a unified shape model. Briefly, homologous or semi-homologous morphological landmarks can be applied to a population of comparable images, which provide the raw data for multivariate analyses of shape in developmental and evolutionary contexts [12]. Though these methods have been widely applied and well developed in zoology and related fields, botanists have only begun to adopt them in studies of leaf and floral shape [13]. Floral morphometric variation has been studied in single plant species to address developmental and evolutionary questions [14–19], and across broader sets of species for taxonomic and evolutionary purposes [20–23]. In this study, we use geometric morphometrics to compare and categorize the diverse floral forms in a charismatic clade, Core Goodeniaceae.

The Goodeniaceae is a species-rich family that currently includes more than 420 species across 12 genera [24], with Australia being the centre of diversity. The family is circumscribed into two clades, LAD (*Lechenaultia*, *Anthotium*, and *Dampiera*) and the larger Core Goodeniaceae ([25]: *Brunonia australis*, and sister clades *Scaevola* s.l. (*Scaevola* sensu stricto with embedded monotypic *Diaspasis filifolia*) and *Goodenia* s.l. (*Goodenia*, *Coopernookia*, *Velleia*, *Verreauxia*, *Selliera*, *Scaevola collaris*, and *Pentaptilon*)). Species in Core Goodeniaceae differ in corolla shape and size, petal placement, color, tactile guides, amount of extra-petal “wing” tissue, morphology of the indusium (appendage of the style for secondary pollen presentation unique to the family), and pollinator rewards. A recent molecular phylogenetic study of the family [26] has also provided evidence that within Core Goodeniaceae there have been multiple independent floral symmetry shifts, most notably in *Goodenia* s.l.

With the exception of one species, all Goodeniaceae have pentamerous, bilaterally symmetrical corollas with a dorsal slit opening the tube of fused petal bases between the two dorsal petals. The depth of the slit varies considerably among species, with the most extreme forms having no fusion of the corolla tube above the ovary. The sole taxon that lacks this slit is the monotypic taxon sister to the remainder of Core Goodeniaceae, *Brunonia australis*, which has been considered the only “true” radially symmetrical Goodeniaceae [27]. However, despite the uniform corolla tube, in our observations the distal petal tips rarely have a radially symmetrical arrangement (S1 Fig). Floral symmetry categorization was included in the descriptions of genera in the taxonomic treatment of the family for *Flora of Australia* [24], but not on a consistent basis. The genus *Scaevola* (commonly named fan-flower) and genus *Selliera* are described as “completely split adaxially”, with all five petals towards the ventral side, resembling a fan. *Goodenia* is described as “usually bilabiate”, and *Velleia* is “bilabiate”. *Diaspasis* and *Coopernookia* are described as “scarcely bilabiate.” At the subgeneric level, there is no mention of the fan-flower form common to all members of *Goodenia* subgenus *Monochila*, nor is there common or consistent discussion of symmetry types for individual species.

The diverse corolla morphologies and putative convergent evolution of forms make the Core Goodeniaceae an excellent system for the study of floral evolution. Floral shape diversity (and thus potentially developmental or evolutionary lability) appears to be greater in *Goodenia* s.l. than sister clade *Scaevola* s.l. Almost all members of *Scaevola* s.l. have fan-flowers where all five petals are opposite the dorsal slit–a form evocative of the ligulate floret of some Asteraceae. All three major clades of *Goodenia* s.l. contain species with fan-flowers, indicating potential convergent evolution of the fan form. There is also extreme diversity in non-fan-flowered morphs, ranging from clearly bilabiate, with dorsal petals nearly 180 degrees apart from lateral petals, to nearly radial, with similar angles between all five petals. *Goodenia* s.l. also includes more species (226 described versus 103 in *Scaevola* s.l.) and is a more ancient lineage than *Scaevola* s.l. (crown ages of 31.7 Ma versus 13.5 Ma; [28]). We ultimately aim to reconstruct floral symmetry evolution across the Core Goodeniaceae to study the tempo of floral divergence and provide complimentary data for detailed taxonomic and evo-devo studies on the group. These efforts are currently limited by uncertainty about the existence or composition of objective categories of floral symmetry in the clade as we rely on the somewhat arbitrary, subjective categories of bilabiate, fan-flowered, and pseudo-radial floral forms, as characterised in the *Flora of Australia*.

Here, we seek to develop and utilize a geometric morphometrics approach that is robust to variation from different photos of individual flowers, captures and describes the major variation in floral symmetry between species, and quantifies and delineates objective floral symmetry categories across the Core Goodeniaceae. Once the robustness of the method to photo variance and potential floral asymmetry and allometric effects is assessed, we apply it across a phylogenetically and morphologically diverse set of Core Goodeniaceae species to test several hypotheses. We hypothesize that the greater variation in floral symmetry form observed in *Goodenia* s.l. rather than *Scaevola* s.l can be quantified. Additionally, we test whether discrete floral symmetry clusters are recovered through geometric morphometrics, and if these correspond to our subjective pseudo-radial, bilabiate, and fan-flower symmetry categorizations. Finally, we explore how independent patterns of morphological variation among the dorsal and lateral petals support the existence of distinct developmental modules in Goodeniaceae floral morphogenesis.

## Materials and Methods

### Image collection

Core Goodeniaceae is a diverse clade with many ephemeral, remote species and narrow range endemics [24]. Rather than accurately describing and differentiating individual or closely related species with extensive intraspecific sampling, the overall goal is to describe floral shape diversity across the breadth of Core Goodeniaceae. To this aim, fewer numbers of floral images are used to represent rarer species than is typical in other geometric morphometrics studies that focus on small numbers of taxa (e.g. [14, 16, 19]). To represent both the phylogenetic diversity and the breadth of floral symmetry morphologies within the group, we captured 335 representative images from 44 species of Core Goodeniaceae from all major clades, as well as one *Dampiera* species from the clade sister to Core Goodeniaceae (Table 1). Images for each species were taken from up to 14 individuals found in multiple populations (7.4 ± 3.3 SD flower images per species). During two collecting trips in southwestern Australia, images of flowers were captured using a digital SLR camera, either in the field or from field-collected living plants maintained in cultivation at Kings Park and Botanical Garden (Perth). For three species not encountered in the field (*Goodenia macmillanii*, *G*. *disperma*, and *G*. *stephensonii*), images from the Atlas of Living Australia (http://bie.ala.org.au/) were used. We aim to be able to include additional images from various sources in subsequent applications of the method, and so wanted to ensure that such publically available data were useable and comparable to photos taken specifically for morphometrics analysis. Flowers were photographed along the axis of the corolla tube, providing a two-dimensional “head-on” view of the corolla. This imaging method provided a consistent, replicable view of the flowers from both field based collections and those downloaded from an online repository.

**Table 1:** Subjective grouping, mean PC scores, k-means clustering, voucher, and collection information for the plants used in this study.

Taxon	Subjective	# pics	PC1 Mean	PC2 Mean	Cluster Mean	k-means	Voucher photographer	Latitude Longitude (GDA 94)	Collection locality (cultivation collection)	Voucher collector	Voucher collection number
*Brunonia australis* R.Br.	Fan	12	0.303	0.004	1.00	fan	S.R. Willis	30° 03′ S 116° 40′ E	Syme Road, east of Wubin, Western Australia	Kelly Shepherd & Spencer Willis	KS 1512
*Coopernookia georgei* Carolin	Bilabiate	1	-0.332	-0.085	2.00	pseudo-radial	A. Gardner	33° 58′ S 119° 48′ E	Fitzgerald River National Park, WNW of Quoin head, Western Australia (Kings Park & Botanic Garden living collection KP20070756)	Max Crowhurst	MCRO 44
*Coopernookia polygalacea* (de Vriese) Carolin	pseudo-radial	5	-0.207	-0.041	2.00	pseudo-radial	S.R. Willis	n/a	Fitzgerald River National Park, Western Australia (Kings Park & Botanic Garden living collection KP19913435)	Luke Sweedman	LSWE 1639
*Coopernookia strophiolata* (Muell.) Carolin	Bilabiate	8	-0.083	0.101	3.00	bilabiate	S.R. Willis	33° 05′ S 119° 42′ E	N on Kathleen Road from the Lake King—Norseman Road, Western Australia	Kelly Shepherd & Spencer Willis	KS 1534
*Dampiera lindleyi* de Vriese	Bilabiate	9	-0.332	-0.129	2.00	pseudo-radial	S.R. Willis	28° 16′ S 114° 30′ E	W on Rob Road from Chilmony Road, NW of Northampton, Western Australia	Kelly Shepherd & Spencer Willis	KS 1515
*Diaspasis filifolia* R.Br.	Bilabiate	13	-0.159	-0.074	2.08	pseudo-radial	S.R. Willis	n/a	n/a (Kings Park & Botanic Garden living collection KP1990618)	Luke Sweedman	LSWE 5049
*Goodenia berardiana* (Gaudich.) Carolin	pseudo-radial	6	-0.088	-0.115	2.00	pseudo-radial	A. Gardner	29° 30′ S 117° 00′ E	Charles Darwin Reserve, Western Australia	n/a	n/a
*Goodenia convexa* Carolin	pseudo-radial	6	-0.242	-0.128	2.00	pseudo-radial	S.R. Willis	n/a	Dandaragan-Badgingarra, Western Australia (Kings Park & Botanic Garden living collection KP20130713)	Luke Sweedman	LSWE 8622
*Goodenia berringbinensis* Carolin	Bilabiate	14	-0.170	0.082	2.79	bilabiate	S.R. Willis	29° 54′ S 120° 31′ E	Ularring Wetland, N of Coolgardie, Western Australia	Kelly Shepherd & Spencer Willis	KS 1531
*Goodenia decursiva* W.Fitzg.	Fan	9	0.190	0.110	1.00	fan	S.R. Willis	33° 54' S 123° 31' E	Mount Pasley, Western Australia (Kings Park & Botanic Garden living collection KP20051416)	Patrick Courtney	PCOU 118
*Goodenia disperma* F.Muell.	Bilabiate	1	-0.240	-0.102	2.00	pseudo-radial	J. Elliott (0513 07)	n/a	Burra Range, Queensland	n/a	n/a
*Goodenia drummondii* Carolin	fan	9	0.314	0.005	1.00	fan	S.R. Willis	28° 13′ S 114° 29′ E	E of Swamp Road, NW of Northampton, Western Australia	Kelly Shepherd & Spencer Willis	KS 1516
*Goodenia filiformis* R.Br.	pseudo-radial	9	-0.129	-0.038	2.22	pseudo-radial	S.R. Willis	29° 50' S 114° 59' 35' E	Coolimba, Western Australia (Kings Park & Botanic Garden living collection KP19920683)	Luke Sweedman	LSWE 1868
*Goodenia hassallii* F.Muell.	bilabiate	9	-0.332	-0.006	2.44	pseudo-radial	S.R. Willis	28° 13′ S 114° 29′ E	E of Swamp Road, NW of Northampton, Western Australia	Kelly Shepherd & Spencer Willis	KS 1517
*Goodenia helmsii* (E.Pritz.) Carolin	fan	9	0.300	-0.012	1.00	fan	S.R. Willis	30° 2′ S 116° 39′ E	N on Manuel Road from the Great Northern Highway, NE of Wubin, Western Australia	Kelly Shepherd & Spencer Willis	KS 1511
*Goodenia macmillanii* F.Muell.	bilabiate	1	-0.366	-0.016	2.00	pseudo-radial	R. Cumming	n/a	n/a (Royal Botanic Garden, Melbourne, Victoria)	n/a	n/a
*Goodenia micrantha* Hemsl. ex Carolin	bilabiate	9	-0.188	0.008	2.56	bilabiate	S.R. Willis	30° 0.4′ S 116° 40′ E	W of Richards Road on the Great Northern Highway, NE of Wubin	Kelly Shepherd & Spencer Willis	KS 1510
*Goodenia mimuloides* S.Moore	pseudo-radial	6*	-0.162	-0.019	2.33	pseudo-radial					
*Goodenia mimuloides* S.Moore		3					S.R. Willis	29° 30′ S 117° 00′ E	N of 7 Mile Well, NE side of Charles Darwin Reserve, Western Australia	Kelly Shepherd & GWG	KS 1550
*Goodenia mimuloides* S.Moore		3					A. Gardner	n/a	NW on Paynes Find—Thundelarra Road from Great Northern Highway, Western Australia	n/a	n/a
*Goodenia occidentalis* Carolin	pseudo-radial	4	-0.221	-0.063	2.25	pseudo-radial	S.R. Willis	29° 30′ S 116° 55′ E	S of Quandong Well, Charles Darwin Reserve, Western Australia	Kelly Shepherd & GWG	KS 1549
*Goodenia ovata* Sm.	bilabiate	10	-0.278	-0.035	2.10	pseudo-radial	S.R. Willis	n/a	n/a (Zanthorrea Nursery, Maida Vale, Western Australia)	Kelly Shepherd & Spencer Willis	KS 1530
*Goodenia phillipsiae* Carolin	bilabiate	4	-0.304	0.207	3.00	bilabiate	S.R. Willis	n/a	n/a (Kings Park & Botanic Garden living collection KP20100889)	Anne Cochrane	
*Goodenia pinifolia* de Vriese	bilabiate	9	-0.145	0.193	2.78	bilabiate	S.R. Willis	31° 50′ S 119° 38′ E	N of the Hyden—Norseman Road on the Marvel Loch—Forrestainia Road, S of Marvel Loch, Western Australia	Kelly Shepherd & Spencer Willis	KS 1532
*Goodenia pusilliflora* F.Muell.	bilabiate	11	-0.240	0.002	2.45	pseudo-radial	S.R. Willis	29° 34′ S 117° 06′ E	Wanarra East Road, W of Great Northern Highway, Western Australia	Kelly Shepherd & GWG	KS 1545
*Goodenia stephensonii* F.Muell.	bilabiate	2	-0.211	0.143	3.00	bilabiate	M. Fagg 20137	32° 24' S 150° 11' E	Murrumbo Gap, 49 km W of Denman, New South Wales (Australian National Botanic Garden, Canberra CBG 52947)	H. Streimann	816
*Goodenia tripartita* Carolin	bilabiate	9	-0.217	-0.069	2.00	pseudo-radial	S.R. Willis	n/a	n/a (Lullfitz Nursery, Wanneroo, Western Australia)	Kelly Shepherd & Spencer Willis	KS 1524
*Goodenia varia* R.Br.	bilabiate	11	-0.304	0.140	3.00	bilabiate	S.R. Willis	32° 43′ S 125° 1′ E	Toolinna Cove, Western Australia (Kings Park & Botanic Garden living collection KP19960634)	Luke Sweedman	LSWE 4419
*Goodenia viscida* R.Br.	fan	7	0.286	0.011	1.00	fan	S.R. Willis	33° 48′ S 120° 10′ E	N of Hopetoun, Western Australia (Kings Park & Botanic Garden living collection KP20050023)	Luke Sweedman	LSWE 6476
*Scaevola anchusifolia* Benth.	fan	11	0.319	0.047	1.00	fan	A. Gardner	26° 10′ S 113° 11′ E	Steep Point, Western Australia (Kings Park & Botanic Garden living collection KP20050795)	Luke Sweedman	LSWE 6585
*Scaevola calliptera* Benth.	pseudo-radial	6*	-0.001	-0.020	1.67	pseudo-radial					
*Scaevola calliptera* Benth.		2					A. Gardner	n/a	n/a (Kings Park & Botanic Garden living collection KP19883190)	n/a	n/a
*Scaevola calliptera* Benth.		2					A. Gardner	31° 39' S 117° 28' E	Tammin, Western Australia (Kings Park & Botanic Garden living collection KP19921313)	Luke Sweedman	LSWE 2368
*Scaevola calliptera* Benth.		2					A. Gardner	n/a	n/a (Kings Park & Botanic Garden living collection KP20000404)	n/a	n/a
*Scaevola canescens* Benth.	fan	5	0.345	-0.040	1.00	fan	A. Gardner	29°47'S 115°15'E	Near Enneaba, Western Australia	n/a	n/a
*Scaevola collaris* J.M.Black ex E.L. Robertson	fan	9	0.376	-0.066	1.00	fan	S.R. Willis	33° 05′ S 119° 35′ E	W of Lake King on the Lake King—Newdegate Road, Western Australia	Kelly Shepherd & Spencer Willis	KS 1533
*Scaevola crassifolia* Labill.	fan	6	0.307	-0.022	1.00	fan	A. Gardner	27°42'S 114° 9'E	Kalbarri beach, Western Australia	n/a	n/a
*Scaevola humifusa* de Vriese	fan	5	0.341	-0.026	1.00	fan	A. Gardner	28° 13′ S 114° 29' E	S on Sandy Gully Road from Rob Road, N of Northampton, Western Australia	Kelly Shepherd & Spencer Willis	KS 1525
*Scaevola phlebopetala* F.Muell.	fan	10	0.150	0.017	1.00	fan	S.R. Willis	28° 01′ S 114° 17′ E	Ogilvie West Road, N of Port Gregory, Western Australia	Kelly Shepherd & Spencer Willis	KS 1519
*Scaevola platyphylla* Lindl.	fan	9	0.302	-0.036	1.00	fan	S.R. Willis	32° 10' S 116° 2' E	Bedfordale, Western Australia (Kings Park & Botanic Garden living collection KP20000831)	Luke Sweedman	LSWE 5356
*Scaevola porocarya* F.Muell.	fan	9	0.258	0.033	1.00	fan	S.R. Willis	28° 11′ S 114° 21′ E	E on Yerina Springs Road from Port Gregory, Western Australia	Kelly Shepherd & GWG	KS 1518
*Scaevola spinescens* R.Br.	fan	11*	0.312	0.025	1.00	fan					
		10					S.R. Willis	30° 03′ S 116° 40′ E	E on Syme Road from Manuel Road, E of Wubin, Western Australia	Kelly Shepherd & GWG	KS 1543
*Scaevola spinescens* R.Br.		1					A. Gardner	29° 30′ S 117° 00′ E	Charles Darwin Reserve, Western Australia	n/a	n/a
*Scaevola thesioides* Benth.	fan	5	0.272	0.005	1.00	fan	A. Gardner	n/a	Eneabba-Leeman, Western Australia (Kings Park & Botanic Garden living collection KP19881040)	Herbert Demarz	HDEM 12029
*Scaevola tomentosa* Gaudich.	fan	10	0.386	-0.030	1.00	fan	S.R. Willis	26° 8' S 113°21' E	Herisson Prong, Shark Bay, Western Australia (Kings Park & Botanic Garden living collection KP19920848)	Luke Sweedman	LSWE 1908
*Selliera radicans* Cav.	fan	1	0.136	0.066	1.00	fan	S.R. Willis	n/a	n/a (Lullfitz Nursery, Wanneroo, Western Australia)	n/a	n/a
*Velleia cycnopotamica* F.Muell.	pseudo-radial	4	0.018	-0.114	1.75	pseudo-radial	S.R. Willis	29° 30′ S 116° 55′ E	S of Quandong Well, Charles Darwin Reserve, Western Australia	Kelly Shepherd & GWG	KS 1548
*Velleia discophora* F.Muell.	bilabiate	5	-0.272	0.213	3.00	bilabiate	S.R. Willis	29° 56′ S 116° 38′ E	E of Burgess Road no Dinnie Road, NE of Wubin, Western Australia	Kelly Shepherd & Spencer Willis	KS 1513
*Velleia foliosa* (Benth.) K.Krause	pseudo-radial	9	-0.265	-0.168	2.00	pseudo-radial	S.R. Willis	n/a	n/a (Kings Park & Botanic Garden living collection KP19990259)	Kelly Shepherd & Spencer Willis	KS 1526
*Velleia rosea* S.Moore	pseudo-radial	8	-0.084	-0.131	2.00	pseudo-radial	S.R. Willis	30° 0.4′ S 116° 40′ E	W of Richards Road on the Great Northern Highway, NE of Wubin, Western Australia	Kelly Shepherd & Spencer Willis	KS 1509
*Verreauxia reinwardtii* (de Vriese) Beth.	bilabiate	9	-0.288	0.070	3.00	bilabiate	S.R. Willis	28° 11′ S 114° 34′ E	N on Chilmony Road from North Road, N of Northampton, Western Australia	Kelly Shepherd & Spencer Willis	KS 1514

For species with multiple populations, the species-level information is on a separate row (total of individual images is denoted with *).

### Morphometrics

**Fig 1:**
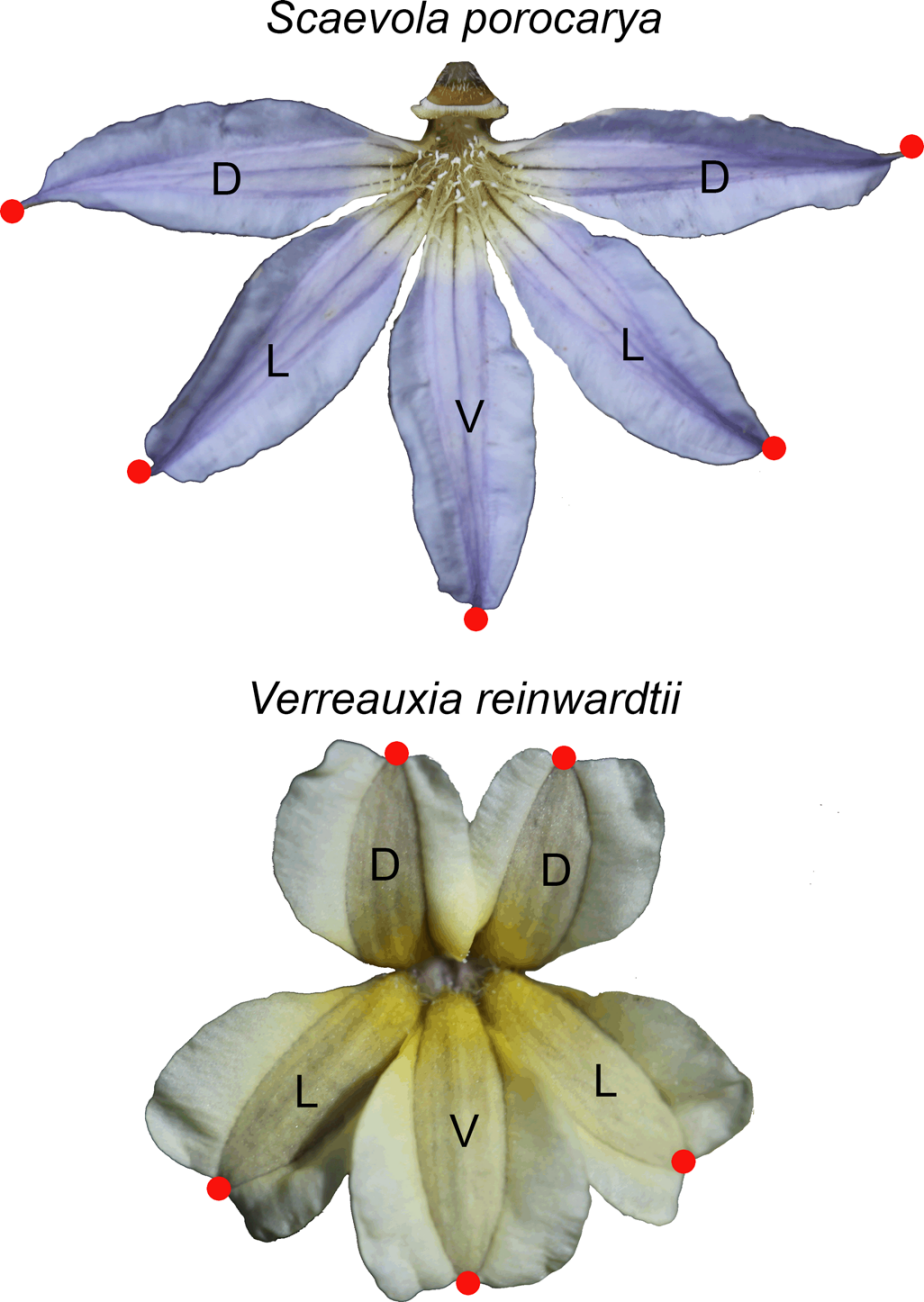
Five point morphometric landmarking scheme for exemplar Core Goodeniaceae taxa. Images of two species (*Scaevola porocarya—*a fan-flower in *Scaevola* s.l., and *Verreauxia reinwardtii—*a bilabiate flower in *Goodenia* s.l.) showing the positions of the 5 landmarks. Dorsal (D), lateral (L), and ventral (V) petals are labelled.

Floral images were landmarked for geometric morphometric analyses using the software tpsDIG2 (available at http://life.bio.sunysb.edu/morph/ [29]). Initially, three different landmarking schemes, using five, 25, and 45 landmarks per image were employed. The five-point scheme placed one clearly homologous landmark at the apex of each of the five petals. The 25-landmark scheme added four landmarks per petal along the margins of the extra-petal wing tissue at axes oriented 90° from positions halfway and three-quarters of the way down the centerline of the visible petal. The 45-landmark scheme added four more landmarks along the margins of the true petals. When petal margins overlapped, landmark positions were estimated based on the distance from the centerline for the opposite landmark. After analysis, each of the three landmark schemes provided similar models for floral morphology. The five-landmark scheme was continued for further study because of its simplicity and reliance on only clearly homologous landmarks (Fig 1).

The x and y coordinates for these five landmarks from each of 335 images were then subjected to Procrustes transformation to minimize scalar and rotational differences, followed by principle components (PC) analysis on their covariance matrix (see [30]) using MorphoJ [31] to distinguish symmetrical vs. asymmetrical contributions to floral morphology. To examine the role of asymmetry in Core Goodeniaceae floral variation, a Procrustes ANOVA was calculated with MorphoJ to ascertain the magnitude of floral shape variance explained by species determination and by asymmetry. Assymetrical components were found to be significant in this data set (p-value = 0.0054; S2 Table) however they accounted for less than 2% of the overall variance. We also examined the influence of assymetry by using centroid sizes (the square root of the sum of squared distances of a set of landmarks from their centroid). If the centroid size of the assymetric component is small compared to symmetric centroid size, and it is uncorrelated to any of the other factors we are studying, we can focus on the symmetric component for downstream analyses and consider assymetries to be a part of the overall environmental error in our calculations. The centroid size of the asymmetrical component averaged 0.1010 (±0.06910 SD), just 4.4% of the symmetrical component (2.305 (±0.368 SD)). ANOVAs calculated on the asymmetric principle components showed no significance with species (p-value = 0.95) or k-means floral shape cluster (p-value = 0.16) as the grouping variable. Finally, a separate analysis of the same landmark data was performed using the PollyMorphometrics 11.1 package on Mathematica [32] that does not separate asymmetric components. Models were consistent with those of the MorphoJ symmetric models. All together, these suggest that the flowers are primarily bilaterally symmetrical and that asymmetries in these populations represent a random variance. In addition to asymmetry we explored potential allometric relationships between shape and size using average corolla lengths by species from *Flora of Australia* [24]. Corolla lengths were not found to correlate with the Procrustes transformed centroid size (p = 0.605, r^2^ = .006) or PC scores (PC1: p = 0.946; PC2: p = .731) by linear regression, and an ANOVA of corolla lengths with k-means floral shape cluster was insignificant (p-value = 0.82; S3 Table). All of these factors suggest that differences among these flowers arise primarily as a consequence of symmetrical variation in corolla shape, rather than as a consequence of asymmetric or allometric factors. No noticeable performance differences were observed between images obtained during this study compared to those from the publicly available *Atlas of Living Australia* database.

The first two PCs accounted for greater than 98% of the total symmetric variation among the landmarks and were retained for all subsequent analysis. To convert PC scores into five-landmark models of flower shape, we used a script in Mathematica similar to [32]. In short, a table of residual values was constructed for the distance from each individual flower marker to the consensus Procrustes landmark position. The eigenvectors of the residual covariance matrix were then found. By multiplying a vector of PC values times the corresponding vectors and adding this to the consensus position, a morphology (series of x-y coordinate values) specific to the PCs was obtained. This method was used to find shape vectors that mapped between average PC values for clade or categorical floral morphologies.

### *Goodenia* s.l. versus *Scaevola* s.l.

The results of the symmetrical principal components analysis were used to quantify the greater floral variation that *Goodenia* s.l. exhibits compared to *Scaevola* s.l. Data from 214 *Goodenia* s.l. flowers (31 species) and 100 *Scaevola* s.l. flowers (12 species) were analysed by ANOVA using JMP 10 (SAS Institute). To test for heterogeneity of variances we used Bartlett’s test [33]. When needed, the non-parametric Wilcoxon rank sum tests of means [34] along with the Levene’s tests of heterogeneity of variances [35] were used to accommodate non-normal distributions. *Brunonia australis* and *Dampiera lindleyi* were excluded from these analyses because they fall outside of these two clades.

### Morphological clustering of flower data

Each species’ flowers were subjectively grouped as fan-flowered, bilabiate, or pseudo-radial. To compare subjective classes with the PCA model, k-means clustering was performed for all 335 individuals with JMP 10, with k = 3 to parallel the number of subjectively assigned morphologies. We used the first two symmetrical principal components to quantitatively describe three distinct sub-populations (following the subjective conceptualization) based entirely on landmark position.

## Results

**Fig 2:**
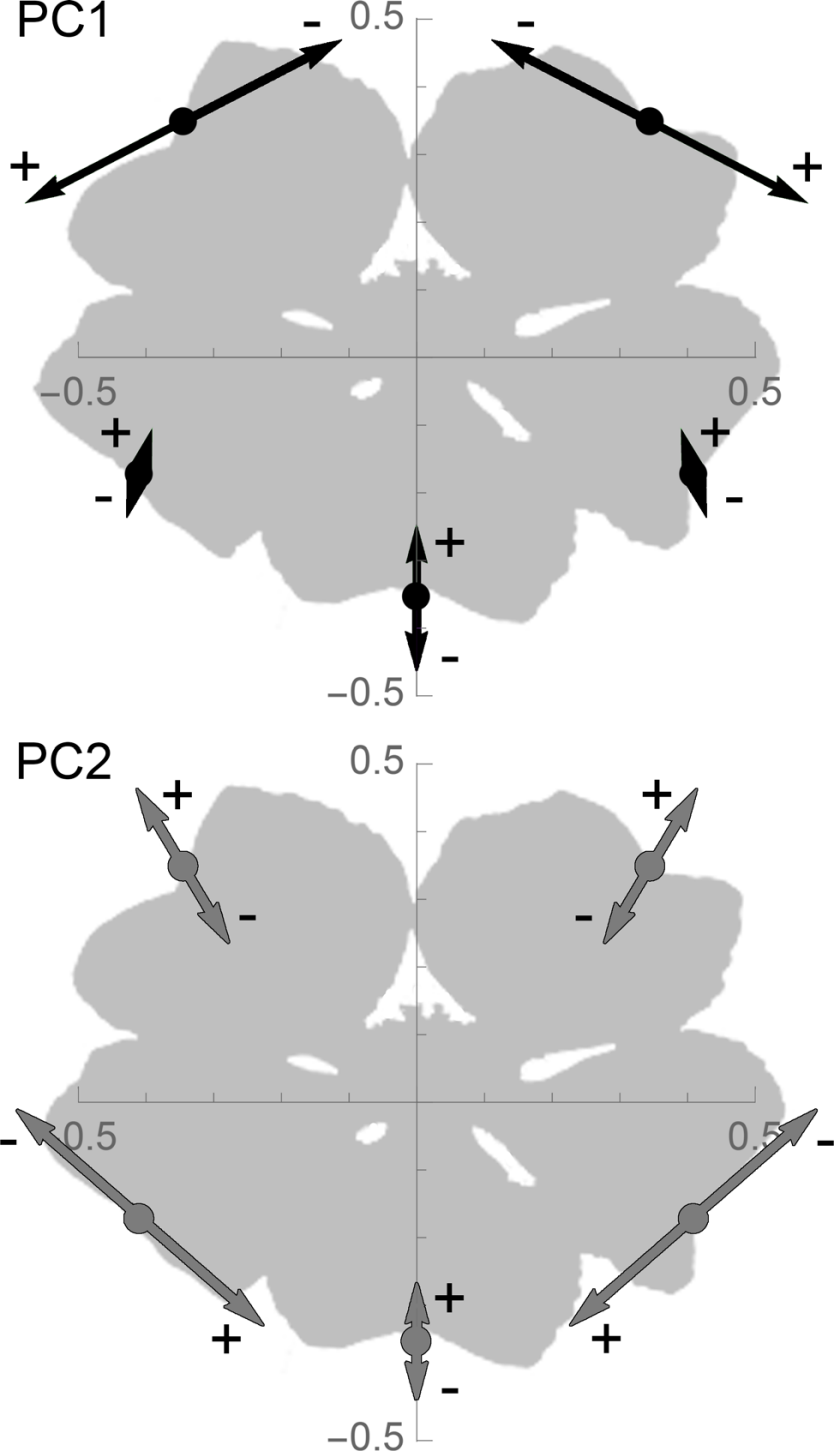
Variation in Core Goodeniaceae floral morphology described by PC1 and PC2. Relative directional influence of the two major principal components of floral landmark variation. The five landmarks are arranged as described by PC scores of zero and zero, with vectors showing shifts associated with PC scores going to ±0.4.

**Fig 3:**
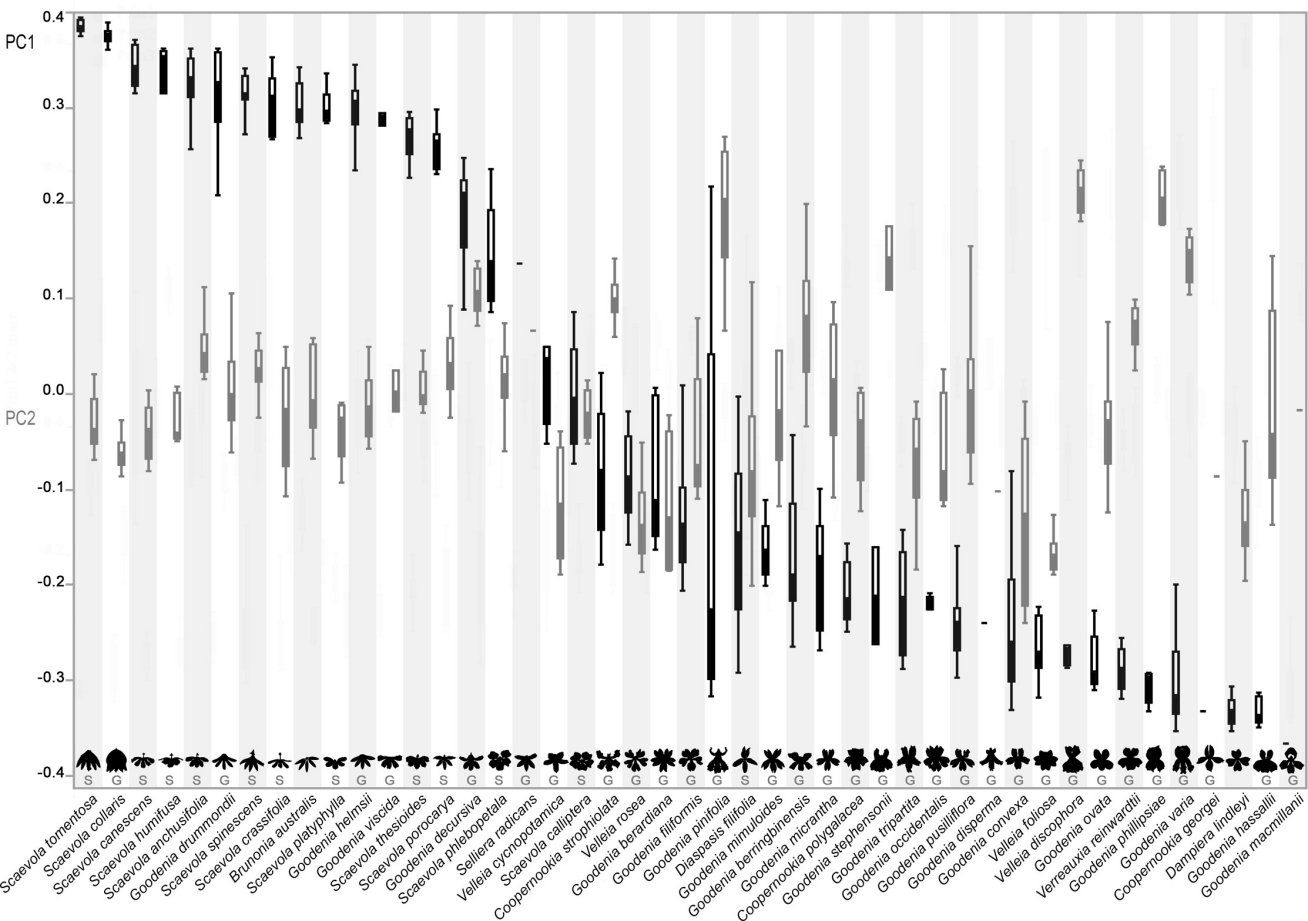
Box plots of variation in 335 PC1 and PC2 scores for 44 Core Goodeniaceae species and *Dampiera lindleyi*. The species are organized by decreasing value of PC1 (black), dorsalizing the dorsal petals (decreasing the angle between them). Increasing values of PC2 (gray) primarily ventralize the lateral petals (decreasing the angle between them). The outline of each flower is shown above clade membership denoted with “G” (*Goodenia* s.l.) or “S” (*Scaevola* s.l.). Boxes denote median values along with quartiles.

Principal components analysis of the landmark data from all individual flowers (n = 335) resulted in two components that explained a cumulative 98.5% of the symmetric component of the total variance (PC1: 86.6%, PC2: 11.9%). PC values were then mapped back onto floral morphology using the eigenvectors of the residual covariance matrix (see Methods). Increasing values of PC1 ventralize the dorsal petals (increasing the angle between them), whereas increasing values of PC2 primarily ventralize the lateral petals (decreasing the angle between them) (Fig 2). The manipulation of these two morphological “axes” alone was sufficient to recreate any of the general floral patterns visible in the Core Goodeniaceae (Fig 3).

### *Goodenia* s.l. versus *Scaevola* s.l.

**Fig 4:**
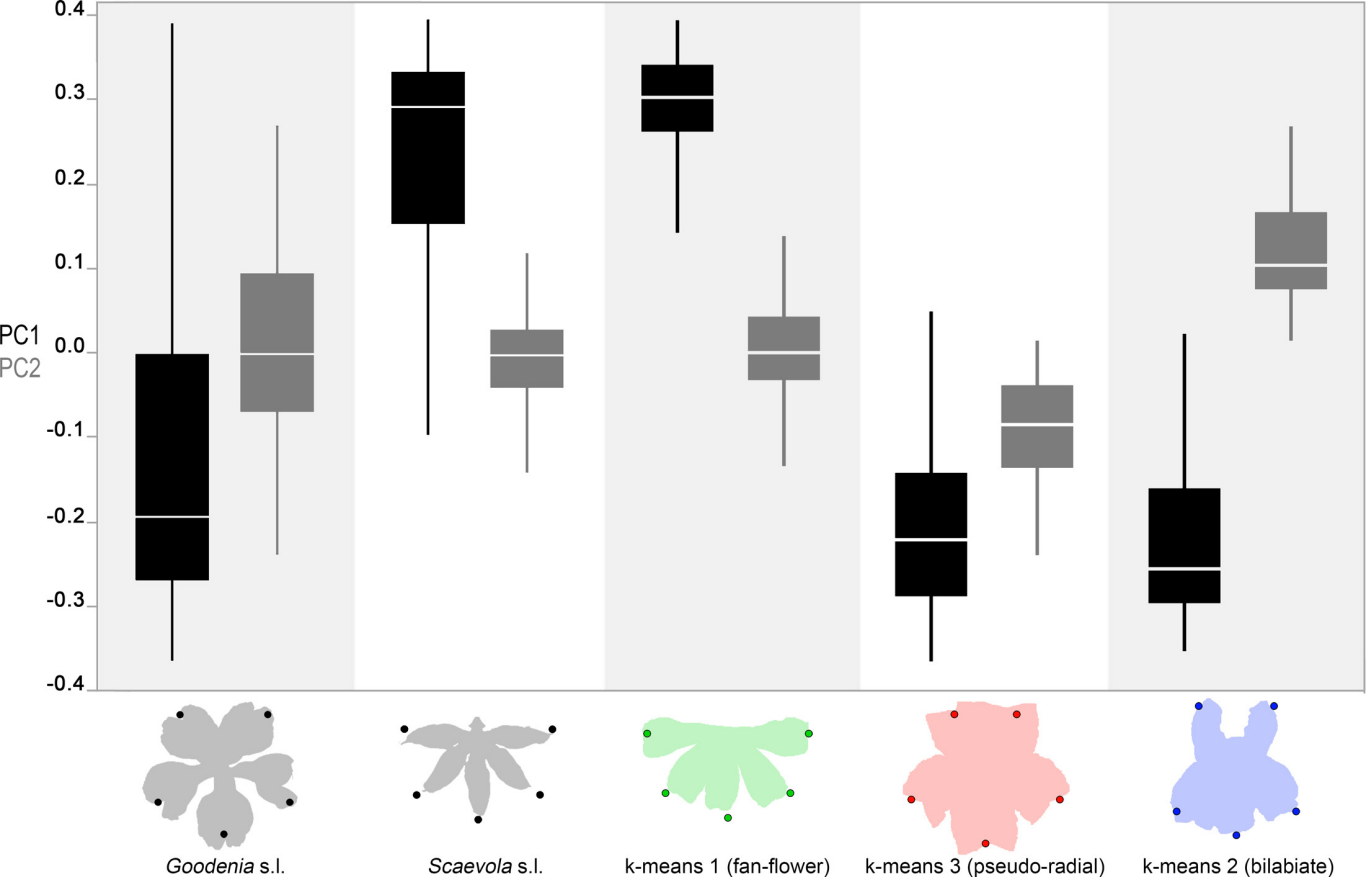
PC1 and PC2 variation in Core Goodeniaceae partitioned by clade and by k-means cluster group. Box plots of PC1 and PC2 scores by clade and by k-means morphological clusters. Boxes denote median values along with quartiles. Below, average landmark positions are superimposed over a flower outline for each group.

When we partitioned images by clade, the members of *Goodenia* s.l. exhibited lower values for PC1 (greater average dorsalization; -0.105 (0.224 SD) on average than those of *Scaevola* s.l. (0.219 (0.179 SD) (Wilcoxon rank sum test p-value <0.0001; Fig 4). The distribution of *Goodenia* in not normal, showing an apparent bimodality on PC1 that includes fan- and non-fan-flowers. Because of this, they have greater variance in PC1 than sister clade *Scaevola* s.l. (Levene’s test p-value = 0.00370). Unlike PC1, PC2 is normally distributed within the floral data. *Goodenia* s.l. has an average PC2 score of 0.00930 (0.112 SD) and *Scaevola* s.l. has and average score of -0.00880 (0.0560 SD). Partitioning the data by clade does not have a significant effect on the differences in mean values (Wilcoxon rank sum test p-value of 0.298), but *Goodenia* s.l. does display an increased variance (Bartlett’s p-value of <0.0001).

### Morphological clustering of species averages

**Fig 5:**
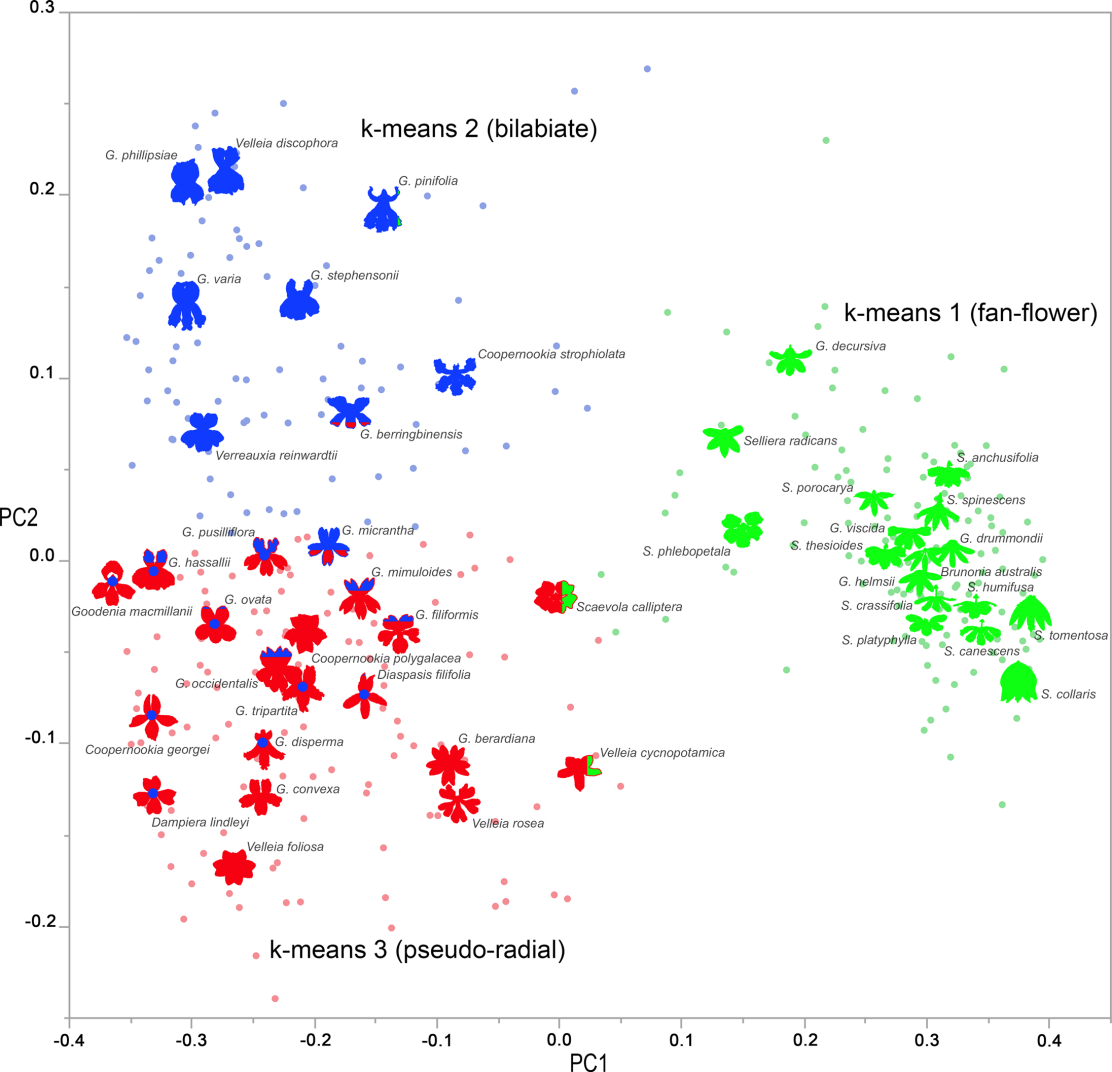
Morphological clustering of Core Goodeniaceae floral diversity. Individuals (dots) and species averages (flower outlines) are depicted in a biplot of their scores for PC1 and PC2 from morphometric analysis. K-means = 1 (fan-flowered) individuals are green, k-means = 2 individuals (bilabiate) are blue, and k-means = 3 (pseudo-radial) individuals are red. Species for which individuals occur in different k-means clusters are illustrated by the proportions of colors depicted in the flower outlines. The flower outline color represents the k-means cluster in the majority, and blue dots indicate when subjective grouping called k-means = 3 (pseudo-radial) species bilabiate.

Geometric morphometrics recovered objective categories of floral symmetry in Core Goodeniaceae (Table 1, Fig 5). All species that had a positive (mean) PC1 value were entirely represented by k-means cluster 1 (fan-flower) with the exception of *Velleia cycnopotamica* (PC1 = 0.0183). This species and *Scaevola calliptera* included three individual flowers clustered in k-means = 1; however, these species average k-means scores were 1.75 (*Velleia cycnopotamica*, n = 4) and 1.67 (*Scaevola calliptera*, n = 6). All individuals with a PC1 value <0 and positive PC2 correlate to the k-means = 2 (bilabiate) cluster, while species with a negative mean PC1 and PC2 were clustered as k-means = 3 (pseudo-radial) flowers, with the exception of *Goodenia pusilliflora* (PC2 = 0.00220). Ten other species have individuals in two different k-means clusters, grading from nearly completely in cluster 2 (bilabiate), through to individuals evenly divided between clusters 2 and 3 (pseudo-radial), to predominantly among cluster 3.

The three clusters delineated by k-means were very similar to the subjective groups, with total agreement between subjective grouping and k-means cluster species averages about the fan-flowers. However, there was significant overlap between k-means cluster 2 and 3 with bilabiate and pseudo-radial flowers respectively. Of the 28 species with PC1 scores less than 0, subjective classes and k-means clusters did not correlate for nine species with PC2 scores around 0. All were subjectively grouped as bilabiate but fell within the “pseudo-radial” k-means cluster 3 (*Coopernookia georgei*, *Dampiera lindleyi*, *Diaspasis filifolia*, *Goodenia disperma*, *G*. *hassallii*, *G*. *macmillanii*, *G*. *ovata*, *G*. *pusilliflora* and *G*. *tripartita*). Both morphometric and subjective methods correlate well with PC1, with adjusted R^2^ values of 88%. For PC2, subjective clustering is less well correlated than k-means, at 20% and 62% respectively.

### Transitions in floral form

When we examine the relative contribution of the PCs to transitions between floral morphologies (Fig 2), it suggests that the PCs represent independent modules in floral morphogenesis. The clear delineation between fan and non-fan-flowers corresponds almost entirely to PC1, with the largest landmark movements in the dorsal petals as they move apart, dorsoventrally. When PC1 is low, pseudo-radial and bilabiate flower shape is described mostly by PC2. As PC2 decreases, bilabiate flowers with lateral petals close to the ventral petal transition to pseudo-radial flowers with widely spread lateral petals. Ultimately, each of these shifts in morphospace sets up potential hypotheses about gene regulation that may pattern these PC shifts.

## Discussion

Floral symmetry diversity in Core Goodeniaceae is highly variable; however, this variation is over simplified by the use of broad subjective terms like fan-flower, bilabiate and pseudo-radial to describe floral form in the group [24]. We aimed to apply a geometric morphometric approach to quantify flower shape in order to better characterize variation in the flowers of Core Goodeniaceae. The five-point landmarking scheme and downstream analyses resulted in two PCs that provide a continuous, 2-dimensional framework to describe aspects of floral morphological variation. The first principle component relates most directly to the dorsal petals and the second component relates most to the lateral petals. When PC1 values (which account for 86.6% of total floral variation) increase, a fan-flower results. Lower values of PC1 decrease the ventralization of the dorsal petals to yield a non-fan-flower, corresponding with a greater range in PC2. Increasing values of PC2 primarily ventralize the lateral petals (decreasing the angle between them), describing bilabiate flowers, whereas decreasing values of PC2 increase the angle between the lateral petals describing pseudo-radial flowers. Together, these capture much of the floral shape diversity of Core Goodeniaceae.

### *Goodenia* s.l. exhibits greater floral variation than *Scaevola* s.l.

If a quantitative measure is to be employed to describe morphology, it must accurately account for observable differences in floral morphologies. *Goodenia* s.l. includes fan-flowered, bilabiate, and pseudo-radially flowered species whereas *Scaevola* s.l. includes representatives that are almost exclusively fan-flowered. We found this observed variation was reflected in our metrics as members of *Goodenia* s.l. exhibit greater variation in both PCs (Fig 4). In PC1, in which the means were significantly different, *Goodenia* s.l. exhibits greater variance and a somewhat bimodal distribution split between six fan-flowered species in addition to the 25 pseudo-radial and bilabiate species. PC2 also exhibits greater variance, which is reasonable considering that the fan-flower morphology (with dorsal petals pushed ventrally toward the lateral petals) inherently limits the variance of PC2.

### Floral symmetry clusters are similar to subjective groups

The subjective labelling of floral morphology as fan-flowered, bilabiate or pseudo-radial comes from our attempt at classifying observable patterns in the location of the five petals. We might expect that any mathematical description of floral morphology would also delineate, or at least describe, these classes. We illustrate a strong, shared delineation in both grouping methods between the 17 fan-flowered species and the 28 species exhibiting non-fan morphologies. When the individuals and species are arrayed in a PC1 vs. PC2 biplot, the fan-flowered species occupy a clearly discrete cluster, strongly supporting at least a binary characterization for ancestral floral symmetry reconstruction. In contrast, the bilabiate and pseudo-radial species occupy a semi- continuous and roughly linear vertical axis from low PC2 scores (most pseudo-radial, e.g. *Goodenia berardiana* or *Velleia foliosa*) to high PC2 scores (most bilabiate, e.g. *Goodenia phillipsiae* or *Velleia discophora*).

Given the continuum in form along the PC2 axis, any attempt at grouping is challenging. Subjective grouping and k-means clustering disagree about where the transition from bilabiate to pseudo-radial flowers occurs (Fig 5 and S2 Fig). Subjective grouping discerns 18 bilabiate species and 10 pseudo-radial species, whereas the k-means clustering overlaps these categories, assembling 9 of the subjective bilabiate species into a single cluster, but mixes the remaining 19 subjective bilabiate and pseudo-radial species into a different group. This is of interest, because it is evident that what we may perceive as a bilabiate or pseudo-radial flower, may depend on an interplay between two PCs (reflecting the relative distance between the dorsal and lateral petals), which we may not readily discern. Disagreement might also be further exacerbated by people’s apparently diminished ability to distinguish differences in the PC2 (positive and negative resulting in bilabiate and pseudo-radial forms respectively), based on the low R^2^ value between its scores and subjective clustering, especially compared to PC1. Additionally, our more restricted perception of pseudo-radial symmetry may have caused us to assign fewer species to that group. Again, these are the types of problems inherent in subjective classification that are not encountered in the mathematical description of clusters, though natural variation among species presents challenges to clustering. Notably, there were 12 species with individuals along the margins of the pseudo-radial cluster, and three species whose individuals split almost evenly between the pseudo-radial and bilabiate clusters (*Goodenia hassallii*, k-means avg = 2.44, n = 9; *G*. *pusilliflora*, k-means avg = 2.45, n = 11; and *G*. *micrantha*, k-means avg = 2.56, n = 9). This suggests that any given species may exhibit variation around some typical mean for PC1 and PC2 from a developmental perspective, without strictly adhering to a preconceived classification of morphology.

The lack of a clear delineation between pseudo-radial and bilabiate morphologies makes it difficult to argue for three morphological character states with the current data. Instead, we may consider that the change in morphology characterized by PC2 may be of a continuous nature. It is possible that the addition of more species to analyses such as these may yield a more bimodal or even trimodal distribution along the PC2 axis, but it may also reinforce the apparent continuum we find among these 28 species. Because of this, phylogenetic reconstruction of the evolution of Core Goodeniaceae flowers would require this character (PC2) to be scored as a continuous trait. Our inability to distinguish additional clusters may also be limited by our morphometric method, especially by the limitations of the “head-on” floral images, as many of the species in the bilabiate cluster have characteristically three-dimensional corollas and recurved dorsal petals. Future work should consider the use of multiple floral views or another method to build 3D geometric morphometric models of these flowers [20], which may reveal additional axes of shape variation.

Another interesting result of these analyses is the fact that much of the potential morphospace is currently unoccupied in the Core Goodeniaceae. This could be due to mechanical, genetic, or selective constraints on Goodeniaceae floral morphological evolution. For example, when PC1 scores are above 1.0 (and the dorsal petals are farther separated), this limits the capacity for movement on the PC2 axis (less space between the dorsal and lateral petals) without overlapping the petals substantially, though we do see some petal overlap in many species, particularly with conspicuous extra-petal wings. Alternatively, there could be an underlying genetic cause, similar to epistasis that restricts the expression of a “PC2” developmental module when a “fan-flower” pathway is active. In either case, if PC2 is restricted in a large portion of the study group, its contribution to phenotypic variance is possibly underestimated, as would normally be the case with an epistatic effect.

### Modularity and potential candidate genes

The independent patterns of morphological variation among the dorsal and lateral petals support the existence of distinct developmental modules in Goodeniaceae floral morphogenesis. Several transcription factor candidate genes studied in other groups may play a role in the transition of floral forms described by both PCs. The most studied across angiosperms are members of the *CYC2* clade of *CYCLOIDEA*-like (*CYC*-like) genes [10, 36–38]. Restriction of expression of these genes to the dorsal region of the corolla has been correlated in many angiosperm groups with a transition to bilateral symmetry. Specifically, in core eudicots, *CYC2* genes independently shift from equivalent dorsoventral expression across the corolla (either ubiquitously expressed or not expressed) in radially symmetric flowers to dorsally restricted expression in bilaterally symmetrical flowers. Data from Dipsacales suggests that the extent of dorsal restriction is correlated with petal location [10, 39], allowing us to hypothesize that a similar pathway could potentially play a role in morphological shifts described by changes in PC1. Closely related to Goodeniaceae, the Asteraceae have undergone multiple duplications of *CYC2* members, with one paralog exclusively expressed in ray florets [40–44]. Changes in expression patterns of *CYC2* clade members have been shown to have an effect on petal growth in both Asteraceae and Brassicaceae [45, 46], suggesting the possibility that they could regulate the PC2 morphological changes. Candidate genes involved in floral symmetry and corolla fusion, could be mapped across a clade of species with quantified morphometric shape in order to more clearly hypothesize how gene changes effect subtler shifts in morphology.

### Conclusions and broader picture

This study describes a relatively simple application of geometric morphometrics for characterizing flower symmetry in the Core Goodeniaceae, which could be applicable to other plant groups. We found that the majority of floral shape variation among the Core Goodeniaceae individuals we sampled is symmetrical, and that variation in the ventralization of the dorsal petals dominates floral shape diversity. It confirms the strong distinction between the fan-flower morphology and all others within the clade. The simplicity, both in construction and interpretation, of the five-landmark model used in this research could argue for its application in other pentamerous angiosperm groups.

Further research is necessary to characterize the genetic factors that modulate these morphological shifts and to what extent the multiple transitions to fan-flowers in this clade [26] have been in parallel. The repeated transitions to fan-flowers in Goodeniaceae may be driven by pollinator selection, however much additional research is necessary to identify the pollinators that may influence them and to characterize Goodeniaceae floral evolutionary dynamics.

## Supporting Information

S1 Fig Core Goodeniaceae floral diversity. Species images are arranged by their scores for the first principal component (PC) of floral variation, which is depicted in the upper boxes. The lower boxes correspond to the second PC scores. Additionally, the results of subjective grouping and k-means clustering are depicted with icons for bilabiate, pseudo-radial, and fan-flowers. When there are two icons, they correspond to subjective grouping (left) and k-means clustering (right). When the methods agreed, the consensus is indicated with a single icon. Following [25 and 26], species depicted in red are included in *Scaevola* s.l., in blue are in *Goodenia* s.l. (note *Scaevola collaris* is placed within this clade), while *Brunonia* and *Dampiera* (depicted in grey) are not in these clades.
(TIF)

S1 Table Raw x,y landmark coordinates for all individuals. (XLSX)

S2 Table Procrustes ANOVA calculated for shape. Data from all images were analyzed with MorphoJ to ascertain the overall variance in shape explained by species and by asymmetries (side) in floral morphology.
(XLSX)

S3 Table Analysis of corolla size variation. Total variance of the average corolla lengths of the 44 species used in this study (source) were analyzed with k-means floral shape cluster as a factor.
(XLSX)
