# Supplementary figures and images for: Characterizing Floral Symmetry in the Core Goodeniaceae with Geometric Morphometrics
Source: PLoS One. 2016 May 5;11(5):e0154736. doi: 10.1371/journal.pone.0154736 (PMC4858217; doi:10.1371/journal.pone.0154736)

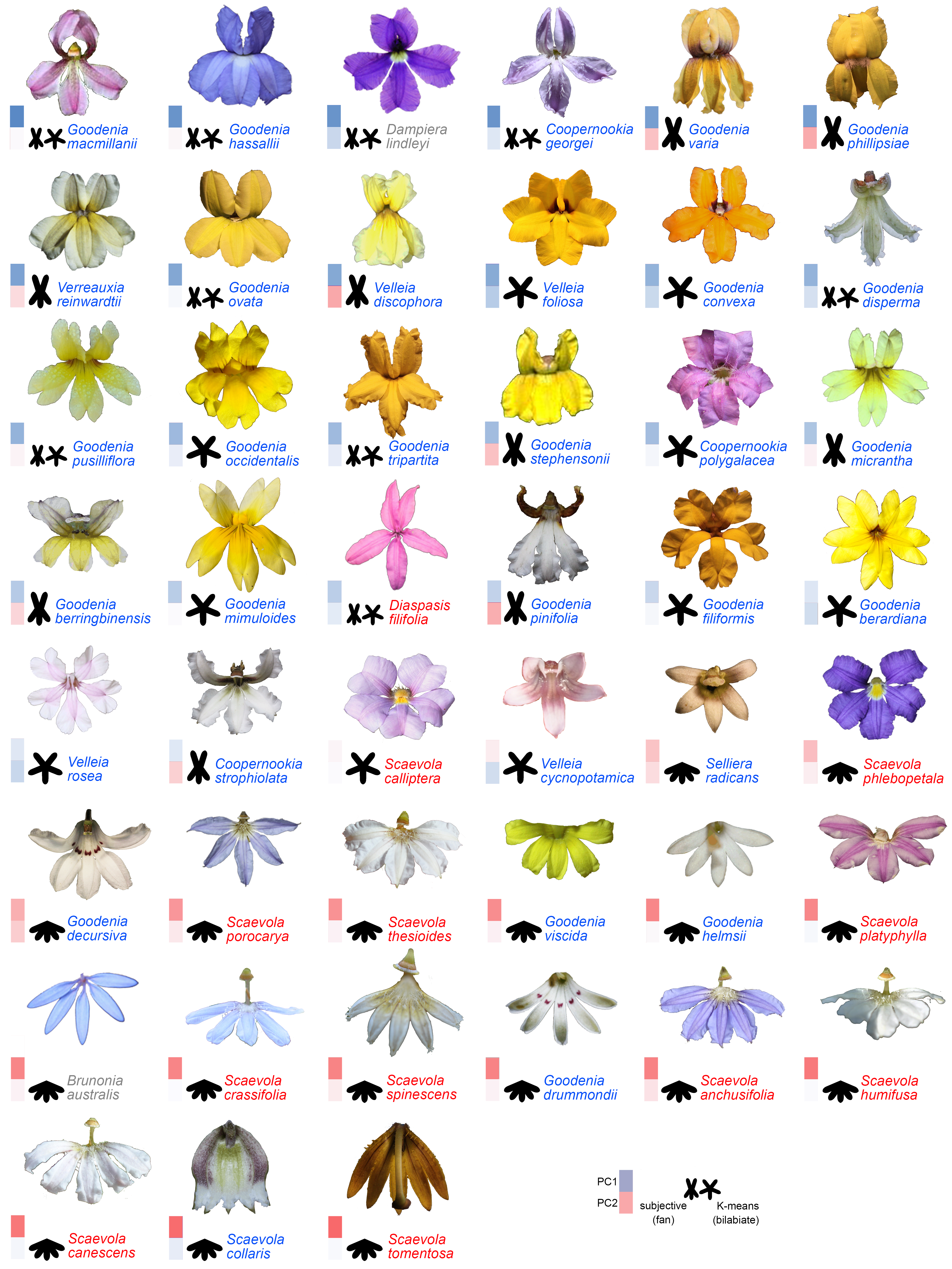

Supplement: S1 Fig — Species images are arranged by their scores for the first principal component (PC) of floral variation, which is depicted in the upper boxes. The lower boxes correspond to the second PC scores. Additionally, the results of subjective grouping and k-means clustering are depicted with icons for bilabiate, pseudo-radial, and fan-flowers. When there are two icons, they correspond to subjective grouping (left) and k-means clustering (right). When the methods agreed, the consensus is indicated with a single icon. Following [25 and 26], species depicted in red are included in Scaevola s.l., in blue are in Goodenia s.l. (note Scaevola collaris is placed within this clade), while Brunonia and Dampiera (depicted in grey) are not in these clades. (TIF) [file pone.0154736.s001.tif]
